# DeepPred-SubMito: A Novel Submitochondrial Localization Predictor Based on Multi-Channel Convolutional Neural Network and Dataset Balancing Treatment

**DOI:** 10.3390/ijms21165710

**Published:** 2020-08-09

**Authors:** Xiao Wang, Yinping Jin, Qiuwen Zhang

**Affiliations:** School of Computer and Communication Engineering, Zhengzhou University of Light Industry, Zhengzhou 450002, China; jyp3215@163.com (Y.J.); 2012032@zzuli.edu.cn (Q.Z.)

**Keywords:** mitochondria, deep learning, imbalance data, mitochondrial intermembrane space

## Abstract

Mitochondrial proteins are physiologically active in different compartments, and their abnormal location will trigger the pathogenesis of human mitochondrial pathologies. Correctly identifying submitochondrial locations can provide information for disease pathogenesis and drug design. A mitochondrion has four submitochondrial compartments, the matrix, the outer membrane, the inner membrane, and the intermembrane space, but various existing studies ignored the intermembrane space. The majority of researchers used traditional machine learning methods for predicting mitochondrial protein localization. Those predictors required expert-level knowledge of biology to be encoded as features rather than allowing the underlying predictor to extract features through a data-driven procedure. Besides, few researchers have considered the imbalance in datasets. In this paper, we propose a novel end-to-end predictor employing deep neural networks, DeepPred-SubMito, for protein submitochondrial location prediction. First, we utilize random over-sampling to decrease the influence caused by unbalanced datasets. Next, we train a multi-channel bilayer convolutional neural network for multiple subsequences to learn high-level features. Third, the prediction result is outputted through the fully connected layer. The performance of the predictor is measured by 10-fold cross-validation and 5-fold cross-validation on the SM424-18 dataset and the SubMitoPred dataset, respectively. Experimental results show that the predictor outperforms state-of-the-art predictors. In addition, the prediction of results in the M983 dataset also confirmed its effectiveness in predicting submitochondrial locations.

## 1. Introduction

Mitochondria are present in almost all eukaryotic organisms. They are usually enclosed by membranes, and their biogenesis is a result of delicate coordination between nuclear and mitochondrial genomes [1]. The mitochondrial intermembrane space is located among two mitochondrial membranes. The mitochondrial matrix is surrounded by the mitochondrial inner membrane [2]. Mitochondria are not only the energy metabolism center of the body, but they also participate in many important cellular pathological processes [3,4], such as electron transfer, adenosine triphosphate synthesis, tricarboxylic acid cycle, fatty acid oxidation, amino acid degradation, and other complex biological processes. Theoretically, for normal cell function, it is critical to have the proteins appear at the right location at the correct time for forming appropriate interactions with correct molecular partners. Mislocalization will make the proteins inaccessible, and thereby not be integrated into the proper functional biological networks or pathways. Dysfunctional mitochondria lead to energy metabolism disorders that cause a series of interacting states of injury. A number of diseases are associated with mitochondria, such as the commonly seen polygenic disorder [5], Parkinson’s disease, diabetes mellitus, etc. Therefore, understanding the protein submitochondrial location can further understand the function of proteins and provide help for the design of auxiliary drugs for diseases caused by mitochondrial defects. Unfortunately, experimental methods to obtain information about the protein submitochondrial location are expensive and time-consuming. It is vital to develop some effective computational methods to assist researchers in solving this problem.

Protein subcellular localization is a significant research area for proteomics, and researchers have acquired some remarkable achievements in recent years. The exploitation of research at the sub-subcellular level is slow, because it is more complicated than that at protein subcellular localization. However, with the increasing amount of sequence data, computational methods suitable for predicting protein submitochondrial location have emerged. Over the last decade, several effective methods achieved distinct achievement in predicting protein submitochondrial location. For example, Mei et al. [6] presented a marked nuclear transfer learning model (MK-TLM) method. Lin et al. [7] employed the Over-Represented Tetrapeptides to predict the submitochondrial location and established the M495 dataset. Kumar et al. [8] put forward a method that could predict the mitochondrial protein location and submitochondrial location. Qiu et al. [9] used pseudo-amino acid composition and pseudo-position-specific scoring matrix to extract features. Yu et al. [10] predicted protein submitochondrial localization by eXtreme gradient boosting. Recently, Savojardo et al. [11] adopted deep learning to predict the four submitochondrial locations.

The prediction of protein submitochondrial localization is a multi-label multi-class problem. It is hard to train a multi-label predictor due to the limitation of the number of proteins with multi-label. In previous multi-class studies, the mitochondrial intermembrane space proteins were always excluded. However, the amount of mitochondrial intermembrane space proteins has increased, and those proteins should be considered in the following research [12]. Among the existing methods, only the methods of Kumar et al. and Savojardo et al. allow the discrimination of four different locations. Thus, it is urgent to propose a novel method to predict the submitochondrial localization including the intermembrane space.

Currently, predicting protein submitochondrial localization methods are mainly based on machine learning algorithms. The traditional machine learning method first requires researchers to extract diverse features from protein sequences, including amino acid composition [13] and pseudo-amino acid composition [14]. After features are transformed into suitable vectors, the vectors are classified [15]. Although those methods have achieved good performance, there still are some essential drawbacks; for example, such manually designed features are very likely to be a suboptimal feature representation. Hence, the performance of models is limited. Compared with machine learning methods that require manual feature extraction, deep learning is a feature learning method that can learn from the original data and classify the abstract features with strong correlation and at a higher level through algorithms. It eliminates the noise of manual intervention. Deep learning has been proven to be a very powerful method that has been successfully applied to various biological applications, including genomics, transcriptome, proteomics, structural biology, and chemistry [15,16,17]. A prediction tool “DeepLoc” [18] based on deep learning was proposed for protein subcellular locations. However, the model considers only one possible label for each protein, whereas the protein subcellular location belongs to a multi-label multi-class problem in general. Long et al. [19] proposed a model combining CNN and XGBoost to solve the problem. Manaz et al. [20] used the CNN model to predict the subcellular localization of endometrial system and secretory pathway proteins. To handle the issue for RNA-protein sequence and structure binding preferences, Pan et al. [21] proposed a model based on convolutional and recurrent neural networks. All of this demonstrates that CNN is an effective deep learning method and widely used in this field.

No predictor is an end-to-end way to predict submitochondrial location. Although Savojardo et al. [11] employed deep learning to predict the submitochondrial location, it also relied on artificial feature extraction. Another problem remains at the subcellular prediction. Rare researchers viewed the matter of skewed data before categorization, which will cause bias for some categories [22,23]. Hence, it is imperative to figure out the classification issue of imbalanced datasets. Convolutional neural networks (CNN) can find motifs in protein sequences, which is very important information for subcellular localization. Therefore, it is very effective to use CNN to capture features in sequences. Unfortunately, CNN cannot capture the effects of past and future states at the current state. To solve this dilemma, we use multi-channel CNN to consider the entire protein sequence.

This paper proposes an end-to-end predictor based on deep learning, namely DeepPred-SubMito. First, it utilizes random over-sampling methods to handle datasets for ensuring the balance among submitochondrial protein classes. Then, it transforms the protein sequence into a one-hot matrix. Finally, it applies multi-channel convolution neural networks to grasp features from protein sequences and output the consequence. We use a cross-validation method to evaluate the performance of our proposed predictor on two datasets containing four submitochondrial locations and compare them with state-of-the-art methods. To further verify the ability of our proposed predictor on a dataset containing only three submitochondrial locations except for the intermembrane space location, we use the M983 dataset to evaluate the performance of our proposed predictor and compare with the state-of-the-art predictors.

The rest of this paper is established as follows. Section 2 discusses the experimental results of DeepPred-SubMito. Section 3 introduces two datasets, random over-sampling, convolutional neural networks, and an evaluation index. Section 4 summarizes this paper.

## 2. Results and Discussion

In this section, we implement the DeepPred-SubMito using Keras [24]. The performance of the proposed predictor was evaluated by testing two submitochondrial datasets, including the SM424-18 and the SubMitoPred. First, we discuss the impact of unbalanced datasets and various deep learning models on performance prediction. Next, performance of the predictor is compared with some excellent methods in the aforementioned datasets.

### 2.1. Parameter Optimization

**Figure 1:**
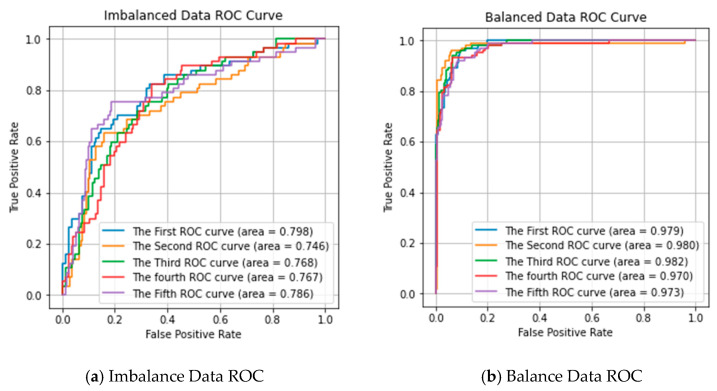
Receiver Operating Characteristic (ROC) curve of our proposed predictor performance in imbalanced data and balanced data. (**a**) Imbalance Data ROC; (**b**) Balance Data ROC.

To validate the effect of random over-sampling on performance prediction, we used the Receiver Operating Characteristic (ROC) curve to estimate our predictor, as shown in Figure 1. Figure 1a shows the multi-class ROC cove of the 5 repeated experiments in imbalanced data. Figure 1b shows the multi-class ROC cove of using the over-sampling method in the dataset. It can observe that the ROC score is significantly improved after using the over-sampling method. The results confirm that the over-sampling approach performs better than without the over-sampling approach.

We set a maximum epoch of 150 and batch size of 64 to explore the effect parameters on the performance of the DeepPred-SubMito predictor. At each stage of the training procedure, we monitor the performance of the training model on the validation dataset. By setting the checkpoint, learning rate, and early stop, the training process will be stopped in advance if the results meet the set prerequisites. Details of hyper-parameter space are summarized in Table 1.

**Figure 2:**
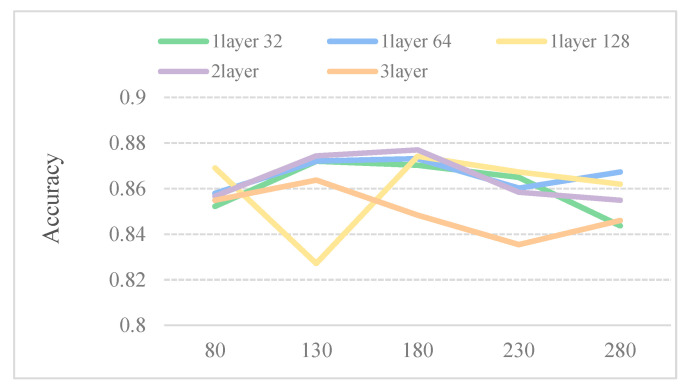
Accuracy of the different sliding window under different convolutional neural networks (CNN) structures on the dataset SubMitoPred.

Considering that the larger the window size W, the lower the number of subsequences, thus, the dataset in such a case is more time consuming. We perform five models to select the optimum parameters for window size W. Table 2 describes five different CNN model structures. As shown in Figure 2, the five predictors perform well when the window size is 180. Therefore, 180 is chosen as the size of sliding window.

**Figure 3:**
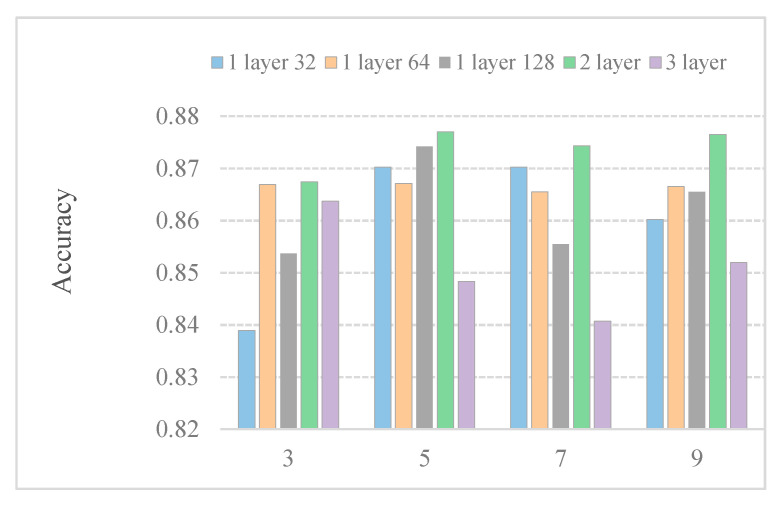
Performance comparison of DeepPred-SubMito with different numbers of CNN structures and kernel sizes in the SubMitoPred dataset.

In general, the convolution kernel size affects feature extraction. To further research the effect of the kernel size on predictor efficiency, we employ predictors with different kernel sizes. Figure 3 shows the accuracy with different kernel sizes. We observe that the kernel size under different models affects the performance of the model differently. The performance of a layer64 model in different convolution sizes is almost the same. When the size of the convolution kernel increases to 7, the performance of a 3-layer model deteriorates. Boosting several kernel sizes does not help much in the five models. When the kernel size is set to 5, the results are the best among the five models. This may be because only part of the features is related to subcellular location.

The research shows that multi-layer CNN can obtain higher-level features [25]. However, with the increase in CNN layers, the computation complexity is higher. To explore the impact of different CNN layers on model performance, we combined different CNN layers with different kernel numbers to process models.

**Figure 4:**
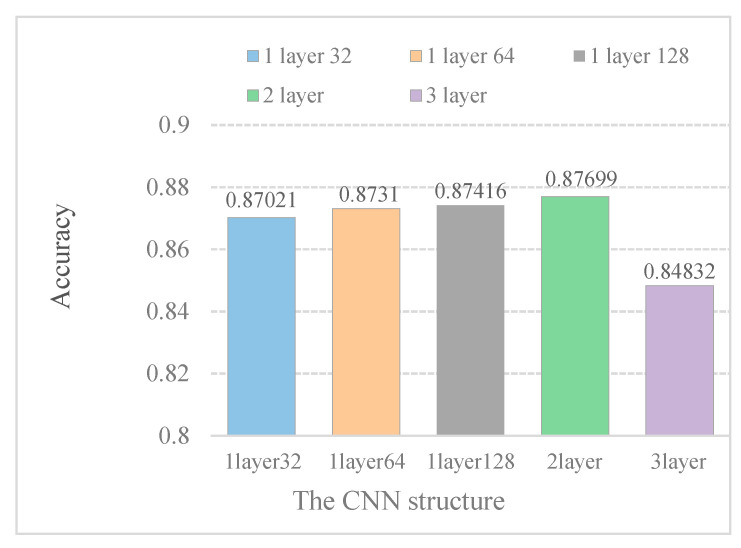
Accuracy of different CNN structure models in the SubMitoPred dataset.

In Figure 4, it is indicated that increasing the kernel numbers can improve model performance. Furthermore, the model performance does not improve with increasing convolution layers. When the DeepPred-SubMito predictor has two convolution layers, the performance is the best. Therefore, we did not construct a too complicated model.

### 2.2. Comparing with Other State-of-the-Art

It is difficult to conduct a uniform comparison of different methods for predicting protein submitochondrial locations because previous studies mainly focused on three positions and rarely considered four positions. Moreover, different methods used different datasets for assessment, many researchers did not provide datasets, and the predictors could not run on their corresponding web servers. For these reasons, this paper only compares the DeepPred-SubMito with DeepMito [11] and SubMitoPred [8] for predicting four protein submitochondria locations. To evaluate the performance of the DeepPred-SubMito predictor for predicting three submitochondrial locations except for the intermembrane space location, we compare it with state-of-the-art predictors on dataset M983.

The DeepMito [11] estimation used the 10-fold cross-validation in the SM424-18 dataset and 5-fold cross-validation in the SubMitoPred datasets. *K*-fold cross-validation is to divide the mitochondrial protein dataset into k subsets, in which one subset of data is used as the testing set and the rest of the *k*-1 subsets of data are used as the training sets. This process is repeated k times, so each subset is used as test data. This paper adopts the same conditions to objectively evaluate the performance of the DeeoPred-SubMito predictor. Table 3 shows prediction results at four locations, respectively.

In the SubMitoPred dataset, the SubMitoPred [8] predictor utilizes the support vector machine (SVM) method to predict protein submitochondrial locations. DeepMito predictor [11] combined features of the position specific scoring matrix (PSSM) and physical–chemical attributes and then used these features to train a single convolution model. Comparing with the SubMitoPred predictor, Matthews Correlation Coefficient values range from 0.46 to 0.65, depending on the compartment. Experiments prove that the deep learning method is effective.

**Figure 5:**
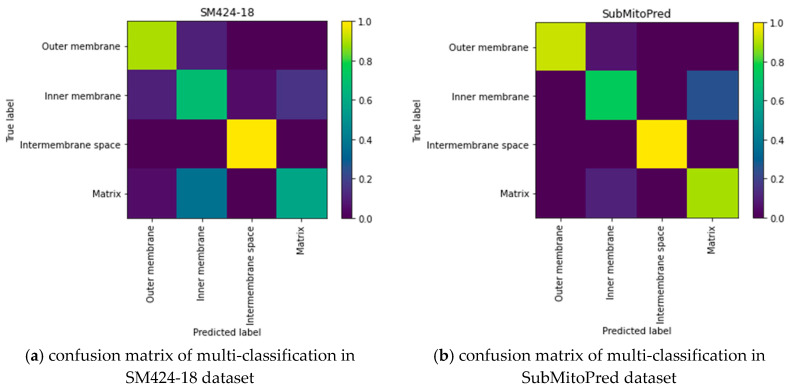
Confusion matrix of multi-classification in SM424-18 and SubMitoPred datasets. (**a**) confusion matrix of multi-classification in SM424-18 dataset; (**b**) confusion matrix of multi-classification in SubMitoPred dataset.

In the SubMitoPred and the SM424-18 datasets, our proposed predictor is better than the DeepMito predictor. From Table 3, we can see that the Matthew Correlation Coefficient (MCC) of the protein submitochondrial was 0.1–0.47 higher than that of the DeepMito predictor. It may be because the DeepMito predictor uses artificially extracted features, missing some useful features. Unlike the DeepMito predictor, our DeepPred-SubMito predictor extracts and classifies features in an end-to-end manner, automatically identifying crucial high-level features. To capture motifs, DeepMito is based on a single-layer CNN architecture. Parallel global average polling and global max polling layers capture different types of patterns. The DeepPred-SubMito predictor extracts and classifies features in an end-to-end manner, automatically identifying crucial high-level features. It eliminates the noise of manual intervention. The DeepPred-SubMito predictor includes the two-layer multi-channel CNN architecture to consider the entire protein sequence feature. To avoid overfitting, the second convolution layer concatenates a dropout layer to randomly remove neurons. In Figure 5, a higher MCC is manifested by a lighter color in the color gradation. It can be seen that the performance for matrix protein is not very good. The DeepPred-SubMito predictor is confused with the inner membrane protein and matrix protein. This may be due to the similarity of protein sequences that make it difficult to distinguish. All in all, the method we proposed is effective in some the existing problems.

To validate the performance of our proposed predictor for predicting three submitochondrial locations except for the intermembrane space location, Table 4 compares the results of our proposed predictor with other predictor methods on the M983 dataset. It can be seen from Table 4 that the DeepPred-SubMito predictor is superior to the SubMito-PSPCP predictor, in which the MCC value on the inner membrane increases by 18%, on the matrix, it increased by 23%, and on outer membrane, it increased by 15%. Compared with the Ahmad predictor, the MCC value at the position of the matrix and outer membrane was 2% and 1.5% lower, but at position of the inner membrane, the MCC value was increased by 7.9%, and the ACC was increased by 2.5%. In contrast to the SubMito-XGBoost predictor, our proposed predictor obtained comparable results. The MCC value on the inner membrane was slightly below 0.5%, but on the other locations, it improved by 0.5% and 2%.

In summary, the DeepPred-SubMito predictor has good prediction results on four and three submitochondrial datasets, which sufficiently indicates that the prediction method constructed in this paper is stable, consistent, and robust.

## 3. Materials and Methods

### 3.1. Datasets

This paper utilizes three datasets, the SM424-18 dataset, the SubMitoPred dataset, and the M983 dataset. The SM424-18 and the SubMitoPred datasets contain four submitochondrial locations. Savojardo et al. [11] established the SM424-18 dataset from UniprotKB/SwissProt (release 2018_02). The author selected full-length proteins with experimental evidence (without fragments). It performed clustering utilizing the CD-HIT program [26] with global alignment and the sequence identity threshold set to 40%. The screened data were 424 mitochondrial proteins. The dataset includes 74 outer membranes, 190 inner membranes, 25 intermembrane spaces, and 135 matrix proteins. Kumar et al. [8] built the SubMitoPred dataset. It comprises 570 mitochondrial proteins, and it is distributed in the four different submitochondrial locations, which include 82 outer membranes, 282 inner membranes, 32 intermembrane spaces, and 174 matrix proteins. The dataset is not screened for a certain species, so the predictor developed in this paper is suitable for the prediction of four submitochondrial positions on all species. The M983 dataset was constructed by Du et al. [27] in 2013. The M983 dataset includes 145 outer membranes, 661 inner membranes, and 177 matrix proteins. The feature of datasets is shown in Table 5.

### 3.2. Sequence Encoding

Convolutional neural networks (CNN) is a classical deep learning model, and it requires the length of each sample in the dataset to be fixed. However, different protein sequences have different lengths. To tackle this problem, the protein sequences are processed by using the following procedure. Initially, the protein sequence with length L is divided into multiple subsequences with length S, each of which is a channel. Therefore, the whole sequence can be divided into (L−S)/(S−W) subsequences with W overlapped shifts. If a protein sequence length is less than S, we fill it with N to a fixed length. After that, the sequence is converted to a one-hot matrix encoding [28,29]. Among them, a protein subsequence $s=\left(s_{1},\ldots ,s_{n}\right)$ is converted into an (n+2m−2)×20 array M, in the following ways:

(1) $$M_{t,f}\{\begin{array}{cc} 0.05 & \mathrm{if}\;\;S_{t-m+1}=N\;or\;t<m\;\mathrm{or}\;t>n-m\\ 1 & \mathrm{if}S_{t-m+1}=f^{\;\;th}\;\mathrm{base}\;\mathrm{in}\;\left(A,R,N,\ldots ,Y,V\right)\\ 0 & \mathrm{otherwise} \end{array}$$

where t is the index of the amino acid, f is the index corresponding to *A*, *R*, *N*, …, *Y*, *N* in the matrix, and m is the size of the convolve filters.

### 3.3. Resolving the Data Imbalance Problem

As mentioned earlier, the mitochondrial subcellular location data shows a high imbalance. The largest ratio between the majority and minority classes reaches approximately 9:1 in the SubMitoPred dataset. In such a case, the predictor is more likely biased [30]. Researchers generally use over-sampling or under-sampling techniques to adjust the multi-class samples. When using the under-sampling to balance the data in this paper, it will discard part of the mitochondrial protein, causing the deterioration of the predictor [31]. Over-sampling is commonly used in deep learning [32,33]. Different from machine learning models, over-sampling cannot result in convolution neural network overfitting [30]. Considering those characteristics, we exploit a random over-sampling algorithm to compensate for the unbalanced data in our algorithm.

Random sampling is the ordinary method in some sampling algorithms. Ling and Li [34] proposed this method, which was proved to be robust. Specifically, it is a strategy to transform the unbalanced sample distribution in the dataset into relative balance. The function of random over-sampling is to randomly generate new minority samples within the group of existing minority outer membrane, inner membrane, matrix protein samples. Suppose $D=D^{1}\cup D^{2}\cup D^{3}\cup D^{4}$ is the sum of the mitochondrial proteins in the four locations. Among them, $D^{1},D^{3},D^{4}$ are the minority sample. Then, we randomly selected samples from the minority to generate new samples $D_{i}^{1},D_{i}^{3},D_{i}^{4}\;\left(i\in n\right)$. The balanced dataset $D_{2}=D_{i}^{1}\cup D^{2}\cup D_{i}^{3}\cup D_{i}^{4}\;\left(i\in n\right)$.

### 3.4. Convolutional Neural Networks

CNN is a multi-layer neural network. The fundamental CNN structure usually includes a convolution layer, activation layer, pooling layer, and full connection layer. The convolution layer is constituted of several convolution kernels, which are used to compute different feature maps. Specifically, the new feature map can be constructed by convolution of the input feature graph with a learned kernel and then applying the nonlinear activation function on the convolution result. Each output feature graph may be the value of combining the convolution of multiple input feature graphs:

(2) $$X_{j}^{l}=f\left(\underset{i\in M_{j}}{\sum }X_{i}^{l-1}*k_{ij}^{l}+b_{j}^{l}\right)$$

where $M_{j}$ represents a selection of input maps, $k_{ij}$ is the convolution kernel used for the connection between the i characteristic graph of input and the j characteristic graph of output, $b_{j}$ is the offset corresponding to the j characteristic graph, and f is the activation function.

A subsampling layer produces down-sampled versions of the input maps [35]. Assume that layer l is the pooling layer and layer l−1 is the convolution layer. Then, the calculation formula of layer f is

(3) $$\delta_{j}^{l}=\beta_{j}^{l+1}\left(f^{\prime }\left(u_{j}^{l}\right){}^{{}^{\circ}}\mathrm{up}\left(\delta_{j}^{l+1}\right)\right)$$

where $f^{\prime }$ represents a derivative of a function, ${}^{{}^{\circ}}$ represents that each element is multiplied, and up( ) represents up-sampling operation.

The sensitivity of each pixel is obtained and the weight is updated.

(4) $$\frac{\partial E}{\partial b_{j}}=\underset{u,v}{\sum }{\left(\delta_{j}^{l}\right)}_{uv}$$

(5) $$\frac{\partial E}{\partial k_{ij}^{l}}=\underset{u,v}{\sum }{\left(\delta_{j}^{l}\right)}_{u,v}{\left(p_{i}^{l-1}\right)}_{uv}$$

where ${\left(p_{i}^{l-1}\right)}_{uv}$ is each patch convolved with $k_{ij}$ when $X_{i}^{l-1}$ is convolved.

When the l layer is the pooling layer, the l+1 layer is the convolution layer, and the sensitivity of a pixel in the l layer is

(6) $$\delta_{j}^{l}=f^{\prime }\left(u_{j}^{l}\right){}^{{}^{\circ}}\mathrm{conv}2\left(\delta_{j}^{l+1},\;\mathrm{rot}180\left(k_{j}^{l+1}\right),{}^{\prime }{\mathrm{full}}^{\prime }\right).$$

In this case, convolution operation is convolution kernel k rotated 180 degrees twice. Since the weight of the pooling layer is fixed, there is no need to calculate a partial derivative.

### 3.5. Illustration of the DeepPred-SubMito

**Figure 6:**
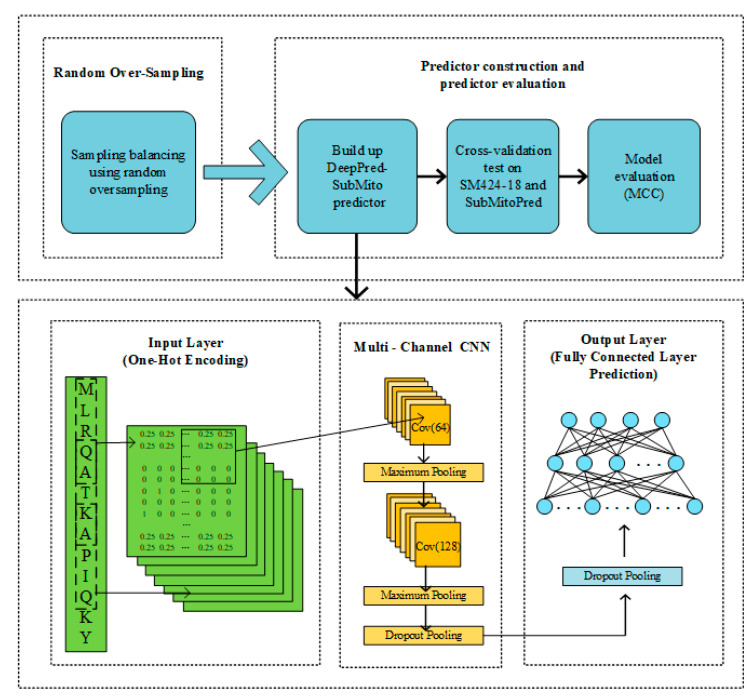
A flowchart of protein submitochondrial localizations prediction based on the DeepPred-SubMito predictor.

For convenience, the protein submitochondrial localization predictor proposed in this paper is called DeepPred-SubMito, and the framework is shown in Figure 6. The detailed steps are as follows:

Step 1: Random Over-sampling. The random over-sampling technology was applied to alleviate the imbalance in datasets. Later, the count of protein sequence of the outer membrane, the inner membrane, the intermembrane space, and the matrix are balanced.

Step 2: Predictor construction and predictor evaluation. A balanced dataset was used to build the predictor. Then, we used 10-fold cross-validation tests on SM424-18 and 5-fold cross-validation tests on SubMitoPred.

Next, we describe the structure of the proposed predictor in detail. Prediction protein submitochondrial localizations can be regarded as a multi-classification problem, in which the input protein sequence pertains to one of four different submitochondrial proteins. The first part is data preprocessing. A sliding window cuts each input protein sequence into the same length subsequence, and then each subsequence is transformed into a one-hot matrix M. The second part includes two convolution layers. Each convolution operation captures features in sequences, and all output of the convolution operation will be concatenated as the input of the subsequent layer. Convolution kernel is employed to scan the input data, and the acquired features are mapped to the activation functions for activation. At last, the data are partitioned and sampled by the maximum pooling layer. The second convolution layer concatenates a dropout layer to randomly remove neurons to avoid overfitting and adjust the number of convolution kernels. Two fully connected layers make up the third part. The first fully connected layer connects a dropout layer. The final fully connected layer has four neurons corresponding to four classifications: the outer membrane, the inner membrane, the intermembrane space, and the matrix.

### 3.6. Evaluation Criteria

To evaluate the performance of the DeepPred-SubMito predictor, accuracy (ACC), Matthews Correlation Coefficient (MCC), and Receiver Operating Characteristic curve (ROC curve) were used as experimental evaluation criteria. The application of the Matthews Correlation Coefficient in multi-classification is called K-categories [36], and it is defined as:

(7) $$\mathrm{MCC}\left(K\right)=\frac{M*S-\sum_{k}^{K}P_{k}*t_{k}}{\sqrt{\left(S^{2}-\sum_{k}^{K}p_{k}^{2}\right)*\left(S^{2}-\sum_{k}^{K}t_{k}^{2}\right)}}$$

where $t_{k}=\sum_{i}^{K}C_{ik}$ is the number of times that class k actually happens, $p_{k}=\sum_{i}^{K}C_{ik}$ is the number of times the k class is predicted, $M=\sum_{k}^{K}C_{kk}$ is the number of correctly predicted samples, and $S=\sum_{i}^{K}\sum_{i}^{K}C_{ij}$ is the total number of samples.

The accuracy is defined as:

(8) $$\mathrm{ACC}\left(K\right)=\frac{{\mathrm{TP}}_{K}}{{\mathrm{TP}}_{K}{+\mathrm{FN}}_{K}}$$

where ${\mathrm{TP}}_{K}$ and ${\mathrm{FN}}_{K}$ are the numbers of true positives, true negatives, and false negatives of the Kth location, respectively [37,38]. Due to ACC having some limitations, we also use the ROC curve, which is a plot of the false positive rate to the true positive rate for all possible prediction thresholds. It is also used to compare the performance of predictors trained on imbalanced datasets [22]. For the above measurement indexes, the higher the measurement value, the better the performance prediction.

## 4. Conclusions

This paper proposes an end-to-end predictor for predicting protein submitochondrial locations. For the use of researchers, the source code of the proposed predictor is available on the GitHub site at https://github.com/jinyinping/DeepPred-SubMito.git. The contribution of the predictor is summarized below. (1) It utilizes random over-sampling to deal with data imbalance. (2) Since the CNN requires an input of fixed length information, this model employs a sliding window to divide each protein sequence into multiple subsequences of the same length and then converts the sequence into a two-dimensional vector. (3) The processed data are directly connected with the specially designed CNN framework. The convolutional layer is used to extract protein sequence information. Compared with the machine learning method relying on artificial feature engineering, the CNN model achieves better outcomes. We compare the performance of the DeepPred-SubMito predictor with baseline methods. Experimental results imply that the nominated predictor achieves better performance than existing predictors. We also evaluated the DeepPred-SubMito predictor performance by changing the convolutional kernel size, number, and CNN layers. The results indicate that the appropriate convolution layer is beneficial to improve performance prediction.

Although DeepPred-SubMito has acquired outstanding results in predicting protein submitochondrial locations, there is still some work to be done subsequently. Natural language processing is well applied in text processing, and we can process protein sequences identical to the text to further improve the performance of DeepPred-SubMito.

## Figures and Tables

**Table 1:** DeepPred-SubMito structure parameters.

Parameter	List of Values Evaluated
Sliding window size (W)	80, 130, 180, 230, 280
Max-pooling	2
Number of convolutional motifs (F)	32, 64, 128
Kernel size (k)	3, 5, 7, 9
Droup (D)	0.25
Optimization	Adam

**Table 2:** CNN structure.

Name	Architecture
1 layer32	32 Convolution kernels
1 layer64	64 Convolution kernels
1 layer128	128 Convolution kernels
2 layer	64/128 Convolution kernels
3 layer	64/64/128 Convolution kernels

**Table 3:** Performance comparison of different predictors.

Datasets	Model	MCC (O)	MCC (I)	MCC (S)	MCC (M)	ACC
SM424-18	DeepMito	0.46	0.47	0.53	0.65	NA
	DeepPred-SubMito	0.85	0.49	0.99	0.56	0.79
SubMitoPred	SubMitoPred	0.42	0.34	0.19	0.51	NA
	DeepMito	0.45	0.68	0.54	0.79	NA
	DeepPred-SubMito	0.92	0.69	0.97	0.73	0.88

MCC (O, I, S, M): Matthew Correlation Coefficient of outer membrane, inner membrane, intermembrane space, and matrix localization, respectively. ACC: accuracy. NA: Not available.

**Table 4:** Prediction results for submitochondrial of the M983 dataset.

Dataset	Model	MCC (I)	MCC (M)	MCC (O)	ACC (%)
M983	SubMito-PSPCP	0.77	0.73	0.83	89.01
	Ahmad et al.	0.871	0.986	0.996	0.951
	SubMito-XGBoost	0.9559	0.9595	0.9604	98.94
	DeepPred-SubMito	0.9503	0.9649	0.9807	97.68

MCC (I, M, O): Matthew Correlation Coefficient of inner membrane, matrix, and outer membrane localization, respectively. ACC: accuracy.

**Table 5:** The feature of datasets.

Compartment	SM424-18	SubMitoPred	M983
Outer membrane	74	82	145
Inner membrane	190	282	661
Intermembrane space	25	32	NA
Matrix	135	174	177
Total	424	570	983

NA: Not available.

## Abbreviations

CNN	Convolutional neural networks
MCC	Matthews Correlation Coefficient
ACC	accuracy
PSSM	position specific scoring matrix
ROC curve	Receiver Operating Characteristic curve
SVM	Support vector machine
